# Prevalence of depressive symptoms among children and adolescents in china: a systematic review and meta-analysis

**DOI:** 10.1186/s13034-024-00841-w

**Published:** 2024-11-19

**Authors:** Jia Zhou, Yiang Liu, Jingyao Ma, Zizhao Feng, Jie Hu, Jia Hu, Bin Dong

**Affiliations:** 1 The National Clinical Research Center for Mental Disorders & Beijing Key Laboratory of Mental Disorders, Beijing Anding Hospital & the Advanced Innovation Center for Human Brain Protection, Capital Medical University, Beijing, China; 2 Institute of Child and Adolescent Health, School of Public Health, Peking University Health Science Center, No. 38 Xueyuan Road, Haidian District, Beijing, 100191 China; 3 Menzies Health Institute Queensland, Griffith University, Nathan, QLD 4111 Australia

## Abstract

**Background:**

Depression is a significant health concern among children and adolescents. Previous epidemiological studies on depressive symptoms in this population have yielded inconsistent findings. This study aims to systematically estimate the prevalence of depressive symptoms among Chinese children and adolescents.

**Method:**

A comprehensive literature search was conducted in both English (PubMed, EMBASE) and Chinese (China National Knowledge Infrastructure, WANFANG) databases from their inception until October 15, 2024. This meta-analysis employed a random-effects model to estimate the pooled prevalence of depressive symptoms.

**Results:**

A total of 439 eligible studies, comprising 1,497,524 participants, were included in the analysis. The pooled point prevalence of depressive symptoms among children and adolescents was found to be 26.17% (95% CI 25.00–27.41%), with significant heterogeneity among studies (I^2^ = 100%, p < 0.001). The most commonly used scales were the SDS and CES-D; the SDS reported a higher prevalence (28.80%, 95% CI 26.88–30.85%) compared to the CES-D (24.50%, 95% CI 22.49–26.68%). There was no clear temporal trend in the prevalence of depressive symptoms over time (r = 0.03, P = 0.74). The highest pooled prevalence was observed in high school students (28.23%, 95% CI 25.58–31.15%), followed by undergraduate students (27.72%, 95% CI 25.79–29.79%) and middle school students (24.15%, 95% CI 21.61–27.00%). Among the provinces, Inner Mongolia exhibited the lowest prevalence (18.43%, 95% CI 11.98–28.36%), while Qinghai and Tibet had the highest rates at 54.19% and 47.50%, respectively, although only two and one study were included for these regions.

**Conclusions:**

The detection rate of depressive symptoms in this study aligns closely with global rates for children and adolescents. High school students exhibit a higher prevalence of depressive symptoms compared to other age groups, highlighting the need for effective interventions targeted at this population. There was no clear temporal trend in the prevalence of depressive symptoms. Additionally, the choice of measurement tool is a critical aspect of epidemiological research; standardizing these measurements is essential for enhancing data comparability across studies.

*Trial Registration* International Prospective Register of Systematic Reviews: No. CRD42023455604.

**Supplementary Information:**

The online version contains supplementary material available at 10.1186/s13034-024-00841-w.

## Introduction

According to the 2015 Global Burden of Disease Study, depression is a worldwide mental health condition and a leading cause of disability in children and adolescents [1, 2]. Major depressive disorder (MDD) is a clinical condition characterized by a persistent low mood and loss of interest; it is considered a significant modifiable risk factor for suicide [3]. It is widely recognized that depression in children and adolescents is a serious condition that predicts future depressive episodes and impairs social functioning [4, 5]. Adolescent depression severely threatens academic performance, social engagement, and long-term health outcomes [6]. Adolescents with depressive symptoms, even those not meeting the full criteria for depression, are highly vulnerable to similar health risks [7]. On one hand, adolescent depressive symptoms can cause attention deficits, memory problems, reduced learning and self-care abilities [8], severe psychological inferiority [9], and social withdrawal [10]. In severe cases, social functioning may be lost, and antisocial behaviors such as self-harm, vandalism, or harm to others may occur, placing a significant burden on families and society [11]. These symptoms are also linked to high-risk behaviors such as suicide attempts [5], substance abuse [12], and risky sexual behaviors (e.g., unprotected sex or having multiple sexual partners) [13]. A previous study found that adolescent depressive symptoms increase the risk of behaviors like fighting, suicidal thoughts, smoking, heavy alcohol use, dieting, drug exposure, and excessive [14]. On the other hand, the adverse effects of adolescent depressive symptoms may extend into adulthood [15]. A study following 710 participants found that adolescents aged 14–16 with depressive symptoms were 3.99 times more likely to develop depression, 2.15 times more likely to experience mood disorders, 6.16 times more likely to develop disruptive behavior disorders, and 2.50 times more likely to suffer from generalized anxiety disorder in adulthood [16, 17]. Adolescent depressive symptoms are also associated with lower educational attainment, increased reliance on social welfare, and higher unemployment rates in adulthood [18, 19]. Additionally, A cohort study that tracked 179 adolescents from age 14 to 24 examined the long-term effects of adolescent depressive symptoms. The study revealed that these symptoms lead to poorer relationship and marital functioning, greater social withdrawal, and heightened feelings of loneliness in adulthood [6]. Adolescent depressive symptoms also raise the risk of chronic non-communicable diseases such as cardiovascular disease and obesity [4, 20].

In China, the weighted prevalence of MDD was 2.0% (95% CI 1.9–2.0) among participants aged 6–16 years [21]. A meta-analysis by Xu et al. [22] on MDD prevalence reported a pooled point prevalence of 1.3%, consistent with global data [22]. Additionally, many children and adolescents show depressive symptoms such as sadness, hopelessness, fatigue, and lack of motivation, though they may not yet meet diagnostic criteria for depression [23]. For instance, Li et al. [24] and Rao et al. [23, 32] found a pooled prevalence of depressive symptoms of 15.4% [24] and 19.85% [23] in children and adolescents respectively. Xu et al. [25] reported a pooled prevalence of 17.2% among primary school students [25], Li et al. [26] reported a prevalence of 22.2% among adolescents [26], and Tang et al. reported 24.3% (95% CI, 21.3%-27.6%) among secondary school students in mainland [27]. The global spread of COVID-19 in 2019 posed immense challenges. Preventive measures like school closures and home isolation have significantly decreased students' physical activity, increased screen time, and reduced social interactions, all of which have negatively impacted their mental health, leading to a rise in depression rates [28, 29]. Two meta-analyses on COVID-19’s impact on depressive symptoms reported pooled prevalences of 29% [30] and 22% [31] respectively.

However, current research on the prevalence of adolescent depressive symptoms has several limitations. Nationwide data on depressive symptom prevalence in children and adolescents are unavailable, although regional surveys have been conducted across the country. Most studies rely on cross-sectional surveys or meta-analyses, lacking temporal trend analysis of detection rates. This is particularly relevant considering China's rapid economic growth and evolving social environment since the twenty-first century, making trend analysis on adolescent depression highly valuable. Furthermore, differences in sampling methods, participant age ranges, assessment scales, and socioeconomic factors across studies conducted in various regions of China have resulted in widely varying epidemiological findings [32]. Additionally, research has not been stratified by educational stages, such as upper primary, junior high, senior high, or early university years. Such stratification is critical for identifying target populations and intervention strategies. Moreover, previous studies have not classified findings by depression scales, such as CES-DC or SDS, which are essential for selecting appropriate screening tools. Although some reviews exist, current meta-analyses from China are limited by age group, study period, sample sizes, and geographic coverage. These meta-analyses included between 12 and 51 studies. The number of included studies is significantly lower than the actual number of surveys conducted in China due to stricter inclusion criteria, resulting in the exclusion of many studies.

This study conducted a meta-analysis of epidemiological surveys on adolescent depressive symptoms from January 1988 to October 2024, incorporating studies to the fullest extent possible. The analysis evaluated temporal trends, distribution across educational stages, differences in assessment tools, and variations in prevalence by gender, province, and residence. The aim of this meta-analysis is to provide a comprehensive assessment of depressive symptom prevalence among children and adolescents in China, addressing a key research gap. The findings offer insights into the lack of understanding of potential moderators and the imbalance in identifying depression prevalence in Chinese children and adolescents, which may contribute to the further development of prevention strategies for adolescent depression.

## Method

This systematic review was conducted in accordance with the PRISMA (Preferred Reporting Items for Systematic Reviews and Meta-Analyses) 2020 guidelines [33]. The protocol was registered with the International Prospective Register of Systematic Reviews (PROSPERO, CRD42023455604).

### Data sources and search strategies

Two researchers (LYA and MJY) independently conducted a literature search in both international (PubMed and EMBASE) and Chinese (China National Knowledge Infrastructure and WANFANG Data) databases from their inception to October 15, 2024. The search terms included: "depress*", "child*", "adolescent*", "adolescence", "student*", "youth", "teenager*", "school", "college", "university", "prevalence", "epidemiolog*", "rate", "percentage", "survey", "China", and "Chinese".

### Study criteria

Comprehensive inclusion and exclusion criteria were predefined to ensure an objective screening of papers. Inclusion criteria were: (1) Cross-sectional or cohort studies (using baseline data only) conducted in China involving children and adolescents, aged 6 to 24. Undergraduate students were included based on recommendations by Sawyer et al., who suggested defining adolescence as spanning ages 10–24 to better reflect developmental trajectories and societal perceptions [34]. (2) The primary outcome must be the incidence of depressive symptoms, clearly identified by an established scale. Exclusion criteria were: (1) Systematic reviews, meta-analyses, conference presentations, or letters; (2) Studies with a sample size of fewer than 150 cases; (3) Research on special populations, such as outpatients, inpatients, obese children, or children with physical or other mental illnesses; (4) Qualitative and experimental studies.

In the first phase, titles and abstracts of relevant publications were screened. In the second phase, full-text papers were obtained and analyzed for inclusion suitability. If multiple papers based on the same dataset were published, only the first published paper with complete data was included. Authors were not contacted for further information. All disagreements were discussed and resolved by consensus with a senior researcher (ZJ).

### Data extraction

A standardized data extraction form was developed. Two reviewers (LYA, MJY) independently extracted and cross-checked data from the included full-text articles. The extracted data included study information (e.g., study name, leading author, journal, publication year, geographic location, language, sampling methods, assessment instruments, and cutoff), participant characteristics (e.g., mean age, sex, school grades, urban or rural residence), and data needed to calculate pooled estimates of prevalence (sample size, response rate, and depressive symptom events).

### Quality assessment

An eight-domain questionnaire, widely used in systematic reviews and meta-analyses [35], was utilized to evaluate the quality of studies meeting the full-text inclusion criteria. The domains assessed were: (1) clarity of the target population definition; (2) use of probability sampling or surveying the entire population; (3) response rate of at least 80%; (4) clear description of non-responders; (5) representativeness of the sample; (6) standardized data collection methods; (7) use of validated criteria to measure depressive symptoms; and (8) provision of prevalence estimates with confidence intervals and subgroup details. Each item was scored as 0 (absence of bias) or 1 (presence of bias), generating a summary score indicating overall risk of bias. All studies were independently rated by LYA and MJY, with discrepancies checked by ZJ.

### Statistical analysis

An overall estimate of depressive symptom prevalence was acquired by pooling the data from eligible papers. We used a random effects model to calculate pooled prevalence estimates with 95% CIs. Heterogeneity was assessed using the I^2^ measure. According to the prespecified cutoffs, low heterogeneity was defined as an I^2^ less than 25%, moderate heterogeneity as an I ^2^ between 25 and 75%, and high heterogeneity as an I^2^ more than 75%. Furthermore, a sensitivity analysis was performed with a “leave one-out” approach, in which all studies are removed one at a time to analyze their influence on pooled estimate and heterogeneity.

Stratified analyses were conducted to identify sources of heterogeneity based on: (1) geographic region (province); (2) year of publication; (3) school grades (primary, middle, high school, undergraduate); (4) urban or rural residence; (5) sex; and (6) assessment instrument including CES-D (Center for Epidemiological Studies Depression) Scale, BDI (Beck Depression Inventory), CDI (Children’s Depression Inventory), SDS (Zung Self-Rating Depression Scale), PHQ (Patient Health Questionnaire), KADS (Kutcher Adolescent Depression Rating Scale), DSRSC (Depression Self-rating Scale for Children), and HAMD (Hamilton Rating Scale for Depression).

All analyses were performed using R 3.5.2 (R Foundation for Statistical Computing, Vienna, Austria), with the R package “meta” employed to generate pooled prevalence estimates and 95% CIs.

## Result

### Study characteristics

**Fig. 1:**
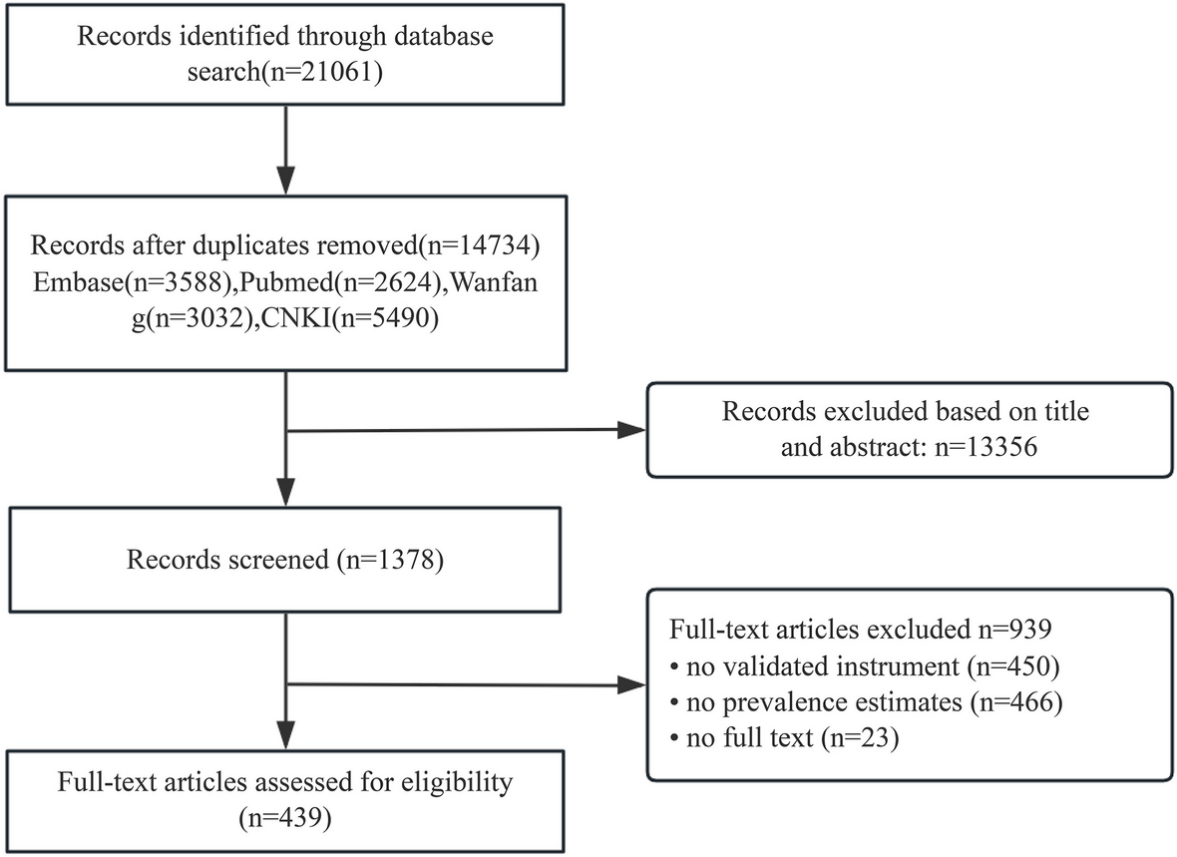
Literature review flowchart

We initially identified 21,061 articles from four academic databases. After removing 6327 duplicates, we assessed the remaining 14,734 articles. A screening of the titles and abstracts resulted in 1378 articles. Following a full-text review, 439 articles that met the inclusion criteria were ultimately included in the analysis. Figure 1 presents a flowchart of the selection process. The publication years spanned 36 years, from 1988 to 2024. The pooled sample size comprised 1,497,524 children and adolescents, with individual sample sizes ranging from 165 to 107,851. The study sites included 33 of the 34 provincial-level administrative regions. Among the 439 included studies, eight different assessment instruments were utilized, namely the BDI, CDI, CES-D, DSRSC, HAMD, KADS, PHQ, and SDS.

### Prevalence of in children and adolescents in China

**Fig. 2:**
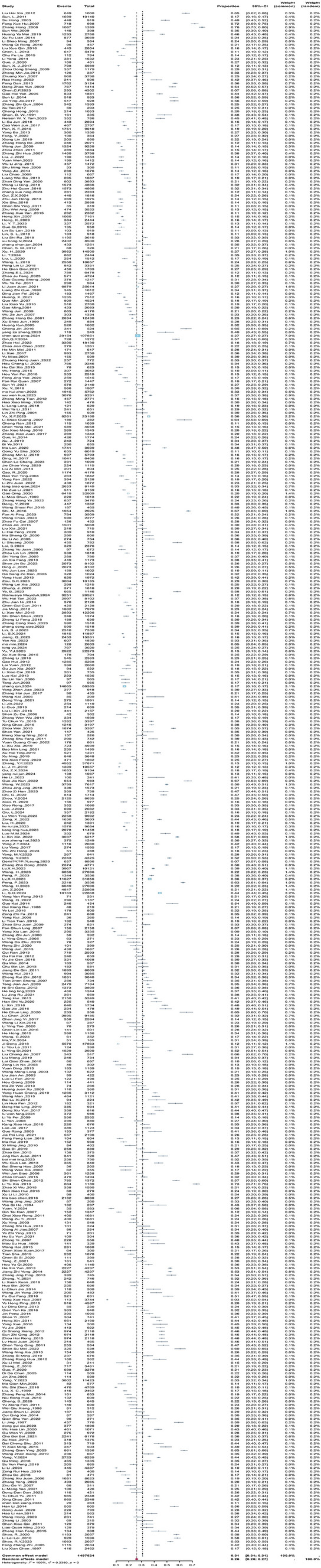
Summary estimates with 95% confidence intervals for the prevalence of depressive symptoms

 The prevalence of depressive symptoms reported in the included studies ranged from 3.0% [36] to 73.2% [37]. The pooled point prevalence of depressive symptoms in children and adolescents was 26.17% (95% CI 25.00–27.41%, I^2^ = 100%, p < 0.001), indicating significant heterogeneity among studies (see Fig. 2).

### Subgroup analyses and meta-regression

The results of the meta-analyses, stratified by sex, grade, and assessment scale, are summarized in Table 1. In this study, the Zung Self-Rating Depression Scale (SDS) and the Center for Epidemiological Studies Depression Scale (CES-D) were the most commonly used instruments. The SDS reported a higher prevalence of depressive symptoms (28.80%, 95% CI 26.88–30.85%), while the CES-D indicated a lower prevalence (24.50%, 95% CI 22.49–26.68%). A significant difference in the prevalence of depressive symptoms between different scales was observed (χ^2^ = 41.89, P < 0.01).

**Table 1:** Subgroup analyses of the prevalence of depressive symptoms in children and adolescents in China

Subgroup	Category	Studies (n)	Sample Size	Proportion [95% CI], %	I^2^	χ^2^	P
Sex	Male	289	1061683	24.68 [23.18–26.28]	99%	1.47	0.23
	Female	296	536032	26.04 [24.53–27.64]	99%		
Grade	Primary school	30	137116	16.97 [14.00–20.56]	99%	26.24	< 0.01
	Middle school	94	342082	24.15 [21.61–27.00]2823 [2558–3115]	100%		
	High school	86	1069851	28.23 [25.58–31.15]	100%		
	Undergraduate school	188	374996	27.72 [25.79–29.79]	100%		
Residence	Urban	97	222191	24.06 [21.82–26.54]2578 [2352–2827]	99%	1.01	0.31
	Rural	98	407379	25.78 [23.52–28.27]	99%		
Scale	SDS	187	1497524	28.80 [26.88–30.85]	99%	41.89	< 0.01
	CES-D	108	549621	24.50 [22.49–26.68]	100%		
	CDI	38	123064	18.27 [16.07–20.78]	99%		
	PHQ	38	302027	27.98 [23.57–33.21]	100%		
	BDI	37	78718	28.56 [23.74–34.34]	100%		
	DSRSC	23	122817	23.90 [20.37–28.04]	100%		
	KADS	4	8236	25.04 [16.81–37.29]	99%		
	HAMD	4	7068	24.36 [15.96–37.16]	99%		

OR [95% CI] for residence is 0.9232 [0.8726; 0.9768], and OR [95% CI] for sex is 0.9280 [0.8879; 0.9698]

**Fig. 3:**
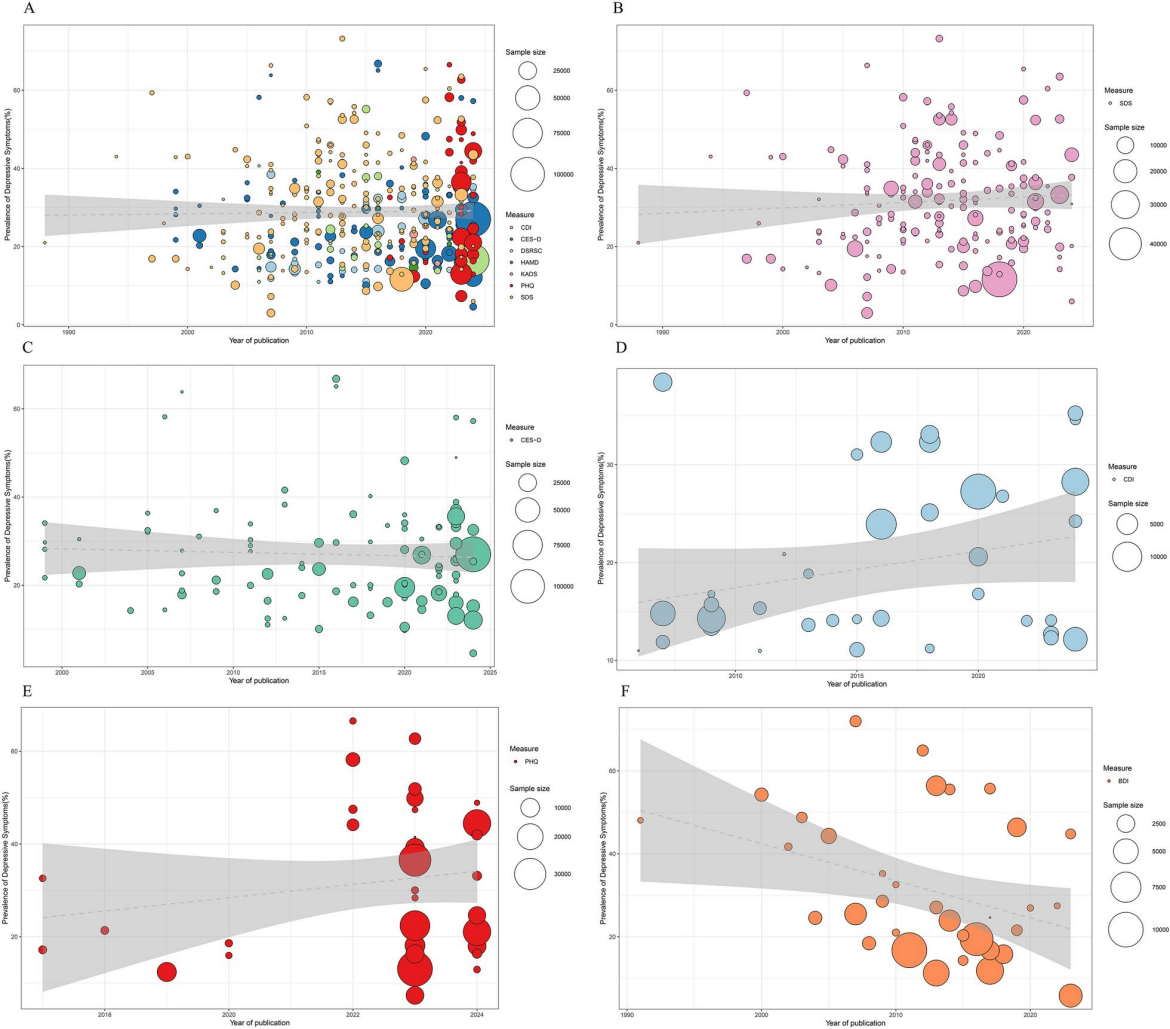
Time trends of depressive symptoms prevalence in children and adolescents assessed with different measures. **A** Depressive symptoms measured using different scales, y = − 35.28 + 0.03x, P = 0.7392. **B** Depressive symptoms measured using the SDS, y = − 246.2 + 0.14x, P = 0.3485, **C** Depressive symptoms measured using the CES-D, y = 186.53 + − 0.08x, P = 0.6259. **D** Depressive symptoms measured using the CDI, y = − 735.48 + 0.37x, P = 0.1206. E. Depressive symptoms measured using the PHQ, y = − 2856.99 + 1.43x, P = 0.3069. F. Depressive symptoms measured using the BDI, y = 1826.71 + − 0.89 x, P = 0.0242

Overall, there was no clear temporal trend in the prevalence of depressive symptoms (r = 0.03, P = 0.74) (see Fig. 3A and Supplementary Table S1). To account for the potential confounding effect of scale choice, an in-depth analysis was conducted that combined scale selection with temporal trends. Studies using different scales exhibited varying trends: the SDS showed an upward trend (r = 0.14), whereas the CES-D indicated a downward trend (r = − 0.08); however, neither trend reached statistical significance (see Fig. 3).

Regarding academic grade, the pooled prevalence of depressive symptoms was highest among high school students (28.23%, 95% CI 25.58–31.15%), followed by undergraduate students (27.72%, 95% CI 25.79–29.79%) and middle school students (24.15%, 95% CI 21.61–27.00%). Primary school students had the lowest prevalence (16.97%, 95% CI 14.00–20.56%). A significant difference in prevalence was found across grades (χ^2^ = 26.24, P < 0.01).

**Fig. 4:**
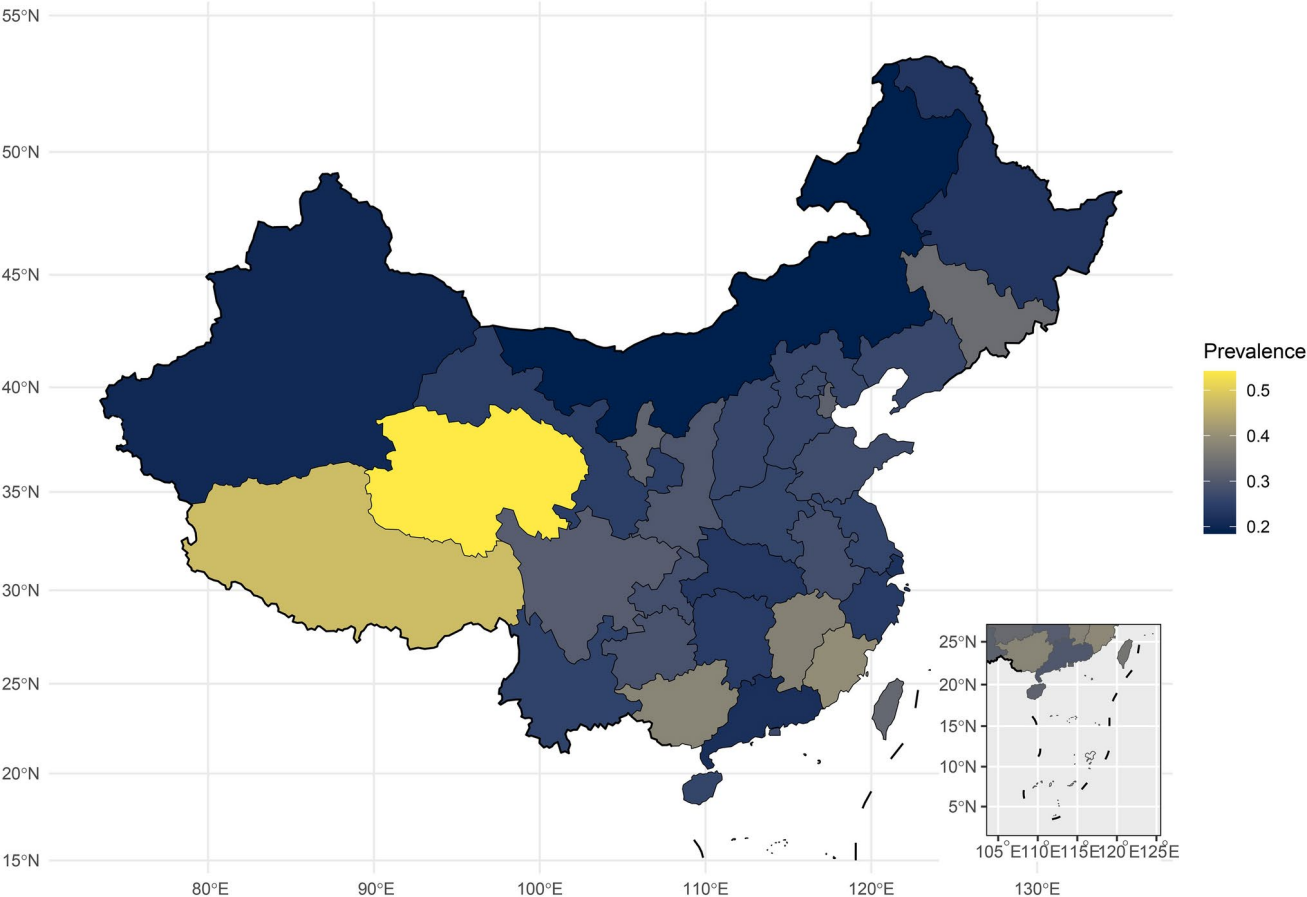
Prevalence of depressive symptoms of children and adolescents in provinces and municipalities. This analysis does not include any studies conducted in Macau. There were 29 articles that did not specify the study location or included multiple provinces, and thus were not included in the map

Among the provinces studied, Inner Mongolia reported the lowest prevalence of depressive symptoms (18.43%, 95% CI 11.98–28.36%). In contrast, Qinghai and Tibet, two plateau regions, exhibited the highest detection rates of depressive symptoms at 54.19% and 47.50%, respectively, although these figures were based on only one or two studies (see Fig. 4 and Supplementary Table S2). Additionally, neither sex nor residence type was significantly associated with the prevalence of depressive symptoms (P > 0.05).

### Quality assessment and publication bias

Sensitivity analysis, which involved sequentially omitting each study, indicated that no single study significantly influenced the overall prevalence estimate of depression (see Supplementary Figure S1). Notably, significant asymmetry was observed in the funnel plot (see Supplementary Figure S2), and substantial publication bias was confirmed through Egger's test (z = − 9.17, P < 0.01) (see Supplementary Figure S3).

Quality assessment scores ranged from 3 to 8, with a median score of 6. All included studies clearly defined their target populations, employed appropriate sampling methods, and utilized validated scales for assessing depressive symptoms, adhering to established criteria. However, only 1.8% (8/439) of studies provided clear descriptions of non-responders. Additionally, prevalence estimates in 32.1% (141/439) of the studies were reported without confidence intervals or subgroup details.

## Discussion

This is an extensive meta-analysis examining the prevalence of depressive symptoms in children and adolescents in China, with 439 studies involving 1,497,524 participants included. In this meta-analysis, we found that the pooled prevalence of depressive symptoms in children and adolescents was 26.17% (95% CI 25.00–27.41%). This pooled prevalence was higher than the 19.85% (95% CI 14.75–24.96%) [23] and 22.2% (95% CI 19.9–24.6%) [26] found in previous relevant meta-analyses. This difference may due to a wider age range of participants in the present study, as the study included primary school students, middle school students, high school students, and undergraduate students. In addition, the pooled prevalence is consistent with the global estimates of child and adolescent depression, as the pooled prevalence estimates of clinically elevated child and adolescent depression was 25.2% [38].

This study found that the detection rate of depressive symptoms was highest among high school students, likely because this group is typically in mid-adolescence, a critical period of physiological and psychological maturation. During this phase, they undergo rapid physical and mental development, face significant academic pressure, experience high parental expectations, and often lack sufficient social experience [39]. Additionally, their hormone levels and the development of the brain's emotion-regulating neural centers remain incomplete, making them more susceptible to psychological, emotional, academic, and interpersonal stress. This stress is exacerbated by high-pressure events such as the National College Entrance Examination (NCEE), or "GaoKao," which is the primary criterion for higher education opportunities [40]. As a result, senior high school students are more likely to experience elevated levels of psychological, emotional, academic, and interpersonal pressure, increasing their risk of depression [40, 41]. These findings are consistent with a previous meta-analysis on the trajectory of depressive symptoms, which revealed that symptoms increase during early adolescence (ages 10–14), peak in mid-adolescence (ages 14–17) during high school, and then decline as individuals transition from late adolescence to early adulthood [42]. Moreover, the study consistently found that the peak in depressive symptoms occurred regardless of group membership, indicating that even adolescents with lower overall levels of depression are susceptible to increased symptoms during this period.

The prevalence of depressive symptoms varied significantly across studies using different scales for assessment. A wide range of depression measurement questionnaires is available. In this study, eight rating scales were included, SDS and CES-D are the most commonly used ones. Consistent with a previous meta-analysis on depressive symptoms in secondary school students, studies utilizing the SDS reported a significantly higher prevalence compared to those using the CDI [27]. The prevalence of depressive symptoms was 18.27% in studies using the CDI, 28.80% in those using the SDS, and 27.98% in those using the PHQ. Additionally, we observed that the three most commonly used scales demonstrated different trends in depressive symptoms over time. This variability may be attributed to the heterogeneity of depression, which presents with a wide range of clinical manifestations [43], such as sadness, insomnia, concentration difficulties, and suicidal ideation. Furthermore, these scales are multidimensional, assessing multiple constructs simultaneously [44]. For example, the SDS, widely used in clinical settings, evaluates four dimensions: psychogenic-emotional symptoms, somatic disorders, psychomotor disturbances, and depressive psychological disorders. The CDI assesses five domains over the past two weeks, including negative mood, interpersonal problems, ineffectiveness, anhedonia, and negative self-esteem. The PHQ-9 consists of nine items assessing the DSM-IV criteria for major depressive disorder, along with an additional item evaluating psychosocial impairment [45]. In summary, the use of different instruments may contribute to the heterogeneity observed in the evaluation of depressive symptoms. Researchers should focus on validating various screening tools. Developing culturally sensitive measures that reflect the socio-cultural context of Chinese children and adolescents is essential.

In this study, boys and girls exhibited similar overall detection rates of depressive symptoms, which contrasts with previous findings. Earlier research indicated that females are twice as likely as males to experience depression, a difference attributed to the sharp rise in depression rates among girls during mid-adolescence [46]. The lack of a gender difference in our study may be due to the wide age range included [26], as this meta-analysis spans from primary school to university-aged individuals. However, gender differences in depressive symptoms vary across age groups. A meta-analysis of 1.9 million individuals from over 90 countries [47] found that gender differences in depression begin to emerge at age 12, peak at age 16, decline by age 19, and stabilize in adulthood. Thus, combining prevalence estimates across age groups may mask these gender differences. Another potential explanation is that the gender gap between Chinese boys and girls may be smaller than in other countries. Research suggests that the depressive trajectories of Chinese boys fall into two subgroups. The majority (85%) experience a gradual increase in depressive symptoms during adolescence (ages 10–19), while the remaining 15% maintain persistently high levels of depression. In contrast, Western boys show a decline in depression during mid-adolescence, while Chinese boys exhibit a steady increase throughout adolescence [27]. The reasons behind this phenomenon remain unclear, and further research is required to explore the role of various risk factors, such as cognitive and interpersonal factors, in shaping this unique gender pattern among Chinese adolescents. Geographic location is another demographic factor that may influence the mental health of children and adolescents, as social resources—including economic support, access to medical care, and educational opportunities—are often unevenly distributed [48]. Additionally, research has shown that higher socioeconomic status and stronger parental educational backgrounds are negatively correlated with depression in offspring [27]. Consistent with previous studies, we observed higher detection rates of depressive symptoms in rural areas compared to urban areas, though the difference was not statistically significant (24.06% vs. 25.78). The findings underscore the urgent need for targeted interventions to strengthen the resilience of children and adolescents in economically disadvantaged regions, such as rural areas, in coping with depressive symptoms [49]. The notably high prevalence rates of depression in plateau regions such as Qinghai and Tibet necessitate further investigation and targeted prevention efforts. This finding aligns with previous studies indicating that the risk of depression increases at higher altitudes [50, 51]. Future studies should examine environmental, social, and cultural factors that may contribute to these disparities, tailoring interventions to specific contexts.

The contribution of this study is multifaceted. First, it provides a comprehensive, nationally representative analysis of depressive symptoms among children and adolescents in China, encompassing a broad temporal and geographical range through the inclusion of 439 studies from 33 provinces, autonomous regions, and municipalities. Second, by identifying the high prevalence of depressive symptoms, particularly among high school students, the study emphasizes the stress-related nature of depression in this population, highlighting key areas for future research and targeted interventions. Moreover, the study underscores the need for improved diagnostic tools, given the heterogeneity observed due to the use of varying scales across the studies. Finally, this research offers crucial insights for policymakers and service providers, aiding the development of effective prevention and treatment strategies for depressive symptoms in children and adolescents. However, some limitations remain. First, the heterogeneity among studies was high, a common challenge in meta-analyses of epidemiological surveys, despite subgroup analyses being conducted. Second, different scales for assessing depressive symptoms were used across the included studies, but no subgroup analyses were performed for different cutoff values of the same scale, potentially contributing to heterogeneity and impacting the results. Third, some studies included in the analysis had a higher risk of bias, with incomplete reporting, such as missing detailed descriptions of non-responders. Fourth, the impact of the COVID-19 pandemic (2020–2023) on mental health outcomes was not explicitly accounted for in the included studies. The pandemic may have altered the context in which depressive symptoms were assessed, potentially affecting the validity and reliability of the findings.

In conclusion, the detection rate of depressive symptoms in this study closely aligns with global rates for children and adolescents. High school students demonstrate a higher prevalence of depressive symptoms compared to other age groups. Notably, detection rates vary significantly depending on the assessment tool used. Among the two most frequently employed scales, the CES-DC yields a lower detection rate that similar to the overall rate. No discernible temporal trend in the detection of depressive symptoms is observed. Substantial regional variations in the prevalence of depressive symptoms among adolescents are evident. For children and adolescents, particularly high school students in rural and plateau areas, there is an urgent need for studies evaluating the effectiveness of various intervention strategies to reduce depressive symptoms.

## Supplementary Information

Additional file 1.

Additional file 2.

Additional file 3.

Additional file 4.

## Data Availability

No datasets were generated or analysed during the current study.
