# Supplementary figures and images for: Prevalence of depressive symptoms among children and adolescents in china: a systematic review and meta-analysis
Source: Child Adolesc Psychiatry Ment Health. 2024 Nov 19;18:150. doi: 10.1186/s13034-024-00841-w (PMC11577650; doi:10.1186/s13034-024-00841-w)

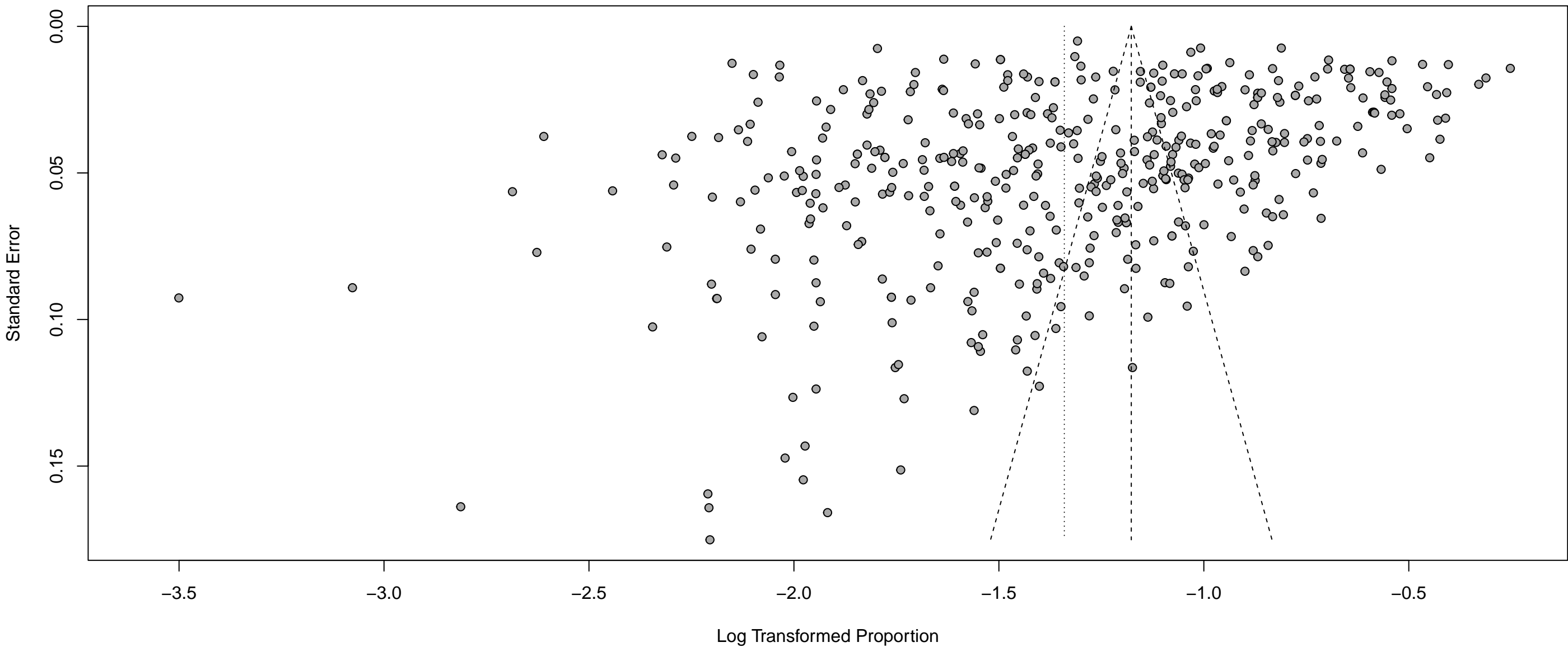

Supplement: Supplementary file 1 — Additional file 1. [file 13034_2024_841_MOESM1_ESM.pdf]

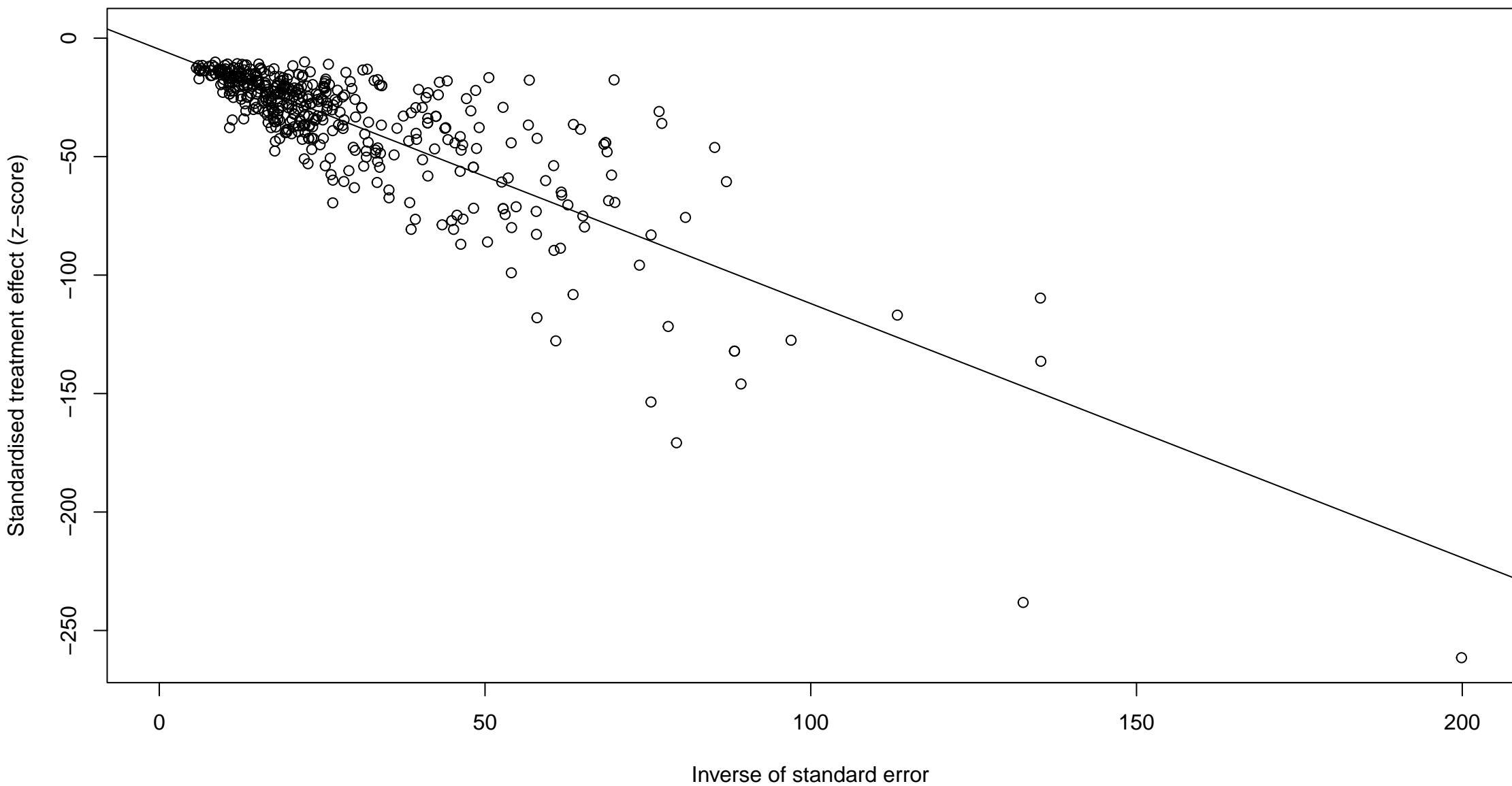

Supplement: Supplementary file 2 — Additional file 2. [file 13034_2024_841_MOESM2_ESM.pdf]
