# Supplementary material for: Prevalence of depressive symptoms among children and adolescents in china: a systematic review and meta-analysis
Source: Child Adolesc Psychiatry Ment Health. 2024 Nov 19;18:150. doi: 10.1186/s13034-024-00841-w (PMC11577650; doi:10.1186/s13034-024-00841-w)
Supplement: Supplementary file 3 — Additional file 3. [file 13034_2024_841_MOESM3_ESM.pdf]

Study	Proportion	95%−CI	P−value	Tau2	Tau	I2
Omitting Liu Hai Xia ,2012	0.26	[0.25; 0.27]	.	0.2367	0.4865	100%
Omitting Sun, L.,2011	0.26	[0.25; 0.27]	.	0.2381	0.4880	100%
Omitting Su Hong ,2003	0.26	[0.25; 0.27]	.	0.2377	0.4876	100%
Omitting Fang Xue Hui,2007	0.26	[0.25; 0.27]	.	0.2362	0.4860	100%
Omitting Zhang Hong ,2008	0.26	[0.25; 0.27]	.	0.2358	0.4856	100%
Omitting Sun Wei,2009	0.26	[0.25; 0.27]	.	0.2384	0.4883	100%
Omitting Huang Ya Mei ,2019	0.26	[0.25; 0.27]	.	0.2378	0.4877	100%
Omitting Xu Fu Lian ,2014	0.26	[0.25; 0.27]	.	0.2386	0.4885	100%
Omitting Li Shao Ming ,2007	0.26	[0.25; 0.27]	.	0.2386	0.4884	100%
Omitting Wang Qi Rong ,2010	0.26	[0.25; 0.27]	.	0.2385	0.4883	100%
Omitting Liu Xue Qin ,2018	0.26	[0.25; 0.27]	.	0.2380	0.4879	100%
Omitting Chen, L.,2013	0.26	[0.25; 0.27]	.	0.2369	0.4867	100%
Omitting Chu Fu Liu ,2015	0.26	[0.25; 0.27]	.	0.2378	0.4876	100%
Omitting Li Yang ,2014	0.26	[0.25; 0.27]	.	0.2386	0.4884	100%
Omitting Guo, J.,2020	0.26	[0.25; 0.27]	.	0.2386	0.4885	100%
Omitting Sun, X. J.,2017	0.26	[0.25; 0.27]	.	0.2371	0.4869	100%
Omitting Zhou Dong Sheng ,2009	0.26	[0.25; 0.27]	.	0.2386	0.4885	100%
Omitting Zheng Min Jie,2010	0.26	[0.25; 0.27]	.	0.2385	0.4884	100%
Omitting Zhuang Xun ,2007	0.26	[0.25; 0.27]	.	0.2386	0.4885	100%
Omitting Rao Hong ,2002	0.26	[0.25; 0.27]	.	0.2381	0.4880	100%
Omitting Feng Dan ,2013	0.26	[0.25; 0.27]	.	0.2372	0.4871	100%
Omitting Dong Zhao Yun ,2000	0.26	[0.25; 0.27]	.	0.2374	0.4872	100%
Omitting Chen,C.P,2023	0.26	[0.25; 0.27]	.	0.2344	0.4841	100%
Omitting Gao Hai Yan ,2005	0.26	[0.25; 0.27]	.	0.2380	0.4878	100%
Omitting Sun Li ,2014	0.26	[0.25; 0.27]	.	0.2373	0.4871	100%
Omitting Jia Ying Jie,2017	0.26	[0.25; 0.27]	.	0.2373	0.4871	100%
Omitting Zhang Zhi Qun ,2004	0.26	[0.25; 0.27]	.	0.2386	0.4885	100%
Omitting Xu Tao,2017	0.26	[0.25; 0.27]	.	0.2386	0.4884	100%
Omitting Chang Hong ,2015	0.26	[0.25; 0.27]	.	0.2384	0.4883	100%
Omitting Chan, D. W.,1991	0.26	[0.25; 0.27]	.	0.2378	0.4876	100%
Omitting Nelson W. Y. Tam,2023	0.26	[0.25; 0.27]	.	0.2379	0.4878	100%
Omitting Li Su Jun ,2018	0.26	[0.25; 0.27]	.	0.2380	0.4879	100%
Omitting Cao Wen Jun ,2017	0.26	[0.25; 0.27]	.	0.2381	0.4880	100%
Omitting Pan, X. F.,2016	0.26	[0.25; 0.27]	.	0.2384	0.4883	100%
Omitting Yang Bo ,2013	0.26	[0.25; 0.27]	.	0.2386	0.4885	100%
Omitting Feng, Y.,2022	0.26	[0.25; 0.27]	.	0.2386	0.4885	100%
Omitting Xiong Lin ,2019	0.26	[0.25; 0.27]	.	0.2385	0.4884	100%
Omitting Zhang Hong Bo ,2007	0.26	[0.25; 0.27]	.	0.2372	0.4870	100%
Omitting Wang Jun ,2009	0.26	[0.25; 0.27]	.	0.2377	0.4876	100%
Omitting Zhou Zhen ,2011	0.26	[0.25; 0.27]	.	0.2379	0.4878	100%
Omitting Zhang Zhi Hua ,2007	0.26	[0.25; 0.27]	.	0.2383	0.4881	100%
Omitting Liu, J.,2022	0.26	[0.25; 0.27]	.	0.2377	0.4875	100%
Omitting Yuan Wen,2023	0.26	[0.25; 0.27]	.	0.2377	0.4876	100%
Omitting Wu Li Jing ,2015	0.26	[0.25; 0.27]	.	0.2385	0.4884	100%
Omitting Shu Ming Yue ,2006	0.26	[0.25; 0.27]	.	0.2371	0.4869	100%
Omitting Yang Jie ,2014	0.26	[0.25; 0.27]	.	0.2377	0.4876	100%
Omitting Liu Chao ,2009	0.26	[0.25; 0.27]	.	0.2381	0.4880	100%
Omitting Liang Wei De ,2013	0.26	[0.25; 0.27]	.	0.2383	0.4882	100%
Omitting Zhan Ding Yan ,2020	0.26	[0.25; 0.27]	.	0.2385	0.4883	100%
Omitting Wang Li Qing ,2018	0.26	[0.25; 0.27]	.	0.2385	0.4884	100%
Omitting Zhu Hui Quan ,2016	0.26	[0.25; 0.27]	.	0.2385	0.4884	100%
Omitting chang xue ning,2023	0.26	[0.25; 0.27]	.	0.2373	0.4871	100%
Omitting Cui, Z.X.2024	0.26	[0.25; 0.27]	.	0.2386	0.4885	100%
Omitting Zhu Jun Hong ,2013	0.26	[0.25; 0.27]	.	0.2376	0.4874	100%
Omitting Xie Shu,2016	0.26	[0.25; 0.27]	.	0.2377	0.4876	100%
Omitting Chen Shi Ying ,2011	0.26	[0.25; 0.27]	.	0.2371	0.4869	100%
Omitting Zhu Wei Ang ,2009	0.26	[0.25; 0.27]	.	0.2376	0.4874	100%
Omitting Zhang Xue Yan ,2015	0.26	[0.25; 0.27]	.	0.2369	0.4867	100%
Omitting Hong Xin ,2007	0.26	[0.25; 0.27]	.	0.2378	0.4877	100%
Omitting Hong, X.,2009	0.26	[0.25; 0.27]	.	0.2380	0.4878	100%
Omitting Li, Y. T.,2023	0.26	[0.25; 0.27]	.	0.2374	0.4872	100%
Omitting Guo Qi,2015	0.26	[0.25; 0.27]	.	0.2377	0.4876	100%
Omitting Lin Su Lan ,2018	0.26	[0.25; 0.27]	.	0.2370	0.4868	100%
Omitting Lin, S. L.,2018	0.26	[0.25; 0.27]	.	0.2370	0.4868	100%
Omitting Liu Shi Ru ,2018	0.26	[0.25; 0.27]	.	0.2385	0.4883	100%
Omitting xu hong lv,2024	0.26	[0.25; 0.27]	.	0.2386	0.4885	100%
Omitting zhang shun jun,2024	0.26	[0.25; 0.27]	.	0.2384	0.4883	100%
Omitting Chan, S. M.,2012	0.26	[0.25; 0.27]	.	0.2385	0.4883	100%
Omitting Xu, H.,2020	0.26	[0.25; 0.27]	.	0.2386	0.4885	100%
Omitting Li, T.2024	0.26	[0.25; 0.27]	.	0.2384	0.4883	100%
Omitting Liu, L.,2020	0.26	[0.25; 0.27]	.	0.2381	0.4880	100%
Omitting Wang, L.,2016	0.26	[0.25; 0.27]	.	0.2386	0.4885	100%
Omitting Peng Lin Li ,2018	0.26	[0.25; 0.27]	.	0.2386	0.4885	100%
Omitting He Qian Qian,2021	0.26	[0.25; 0.27]	.	0.2386	0.4885	100%
Omitting Zhang,E.L.2024	0.26	[0.25; 0.27]	.	0.2372	0.4870	100%
Omitting Zhao Ju Fang ,2023	0.26	[0.25; 0.27]	.	0.2372	0.4870	100%
Omitting Gao Guang Sheng ,2008	0.26	[0.25; 0.27]	.	0.2385	0.4884	100%
Omitting Wu Ya Fei ,2011	0.26	[0.25; 0.27]	.	0.2386	0.4884	100%
Omitting Li Juan Juan ,2021	0.26	[0.25; 0.27]	.	0.2386	0.4885	100%
Omitting Liang Zhi Qun ,1999	0.26	[0.25; 0.27]	.	0.2384	0.4883	100%
Omitting Ding Jian Fei ,2012	0.26	[0.25; 0.27]	.	0.2373	0.4872	100%
Omitting Huang, X.,2021	0.26	[0.25; 0.27]	.	0.2381	0.4879	100%
Omitting Que Min ,2007	0.26	[0.25; 0.27]	.	0.2383	0.4881	100%
Omitting Liu Xiao Yu ,2016	0.26	[0.25; 0.27]	.	0.2383	0.4882	100%
Omitting Gao Ming,2001	0.26	[0.25; 0.27]	.	0.2384	0.4883	100%
Omitting Wang Jun ,2009	0.26	[0.25; 0.27]	.	0.2385	0.4884	100%
Omitting Wu Ze Jun ,2007	0.26	[0.25; 0.27]	.	0.2386	0.4884	100%
Omitting Zhang Hong Bo ,2001	0.26	[0.25; 0.27]	.	0.2386	0.4884	100%
Omitting Xu Shao Jun ,1999	0.26	[0.25; 0.27]	.	0.2386	0.4885	100%
Omitting Huang Kun,2005	0.26	[0.25; 0.27]	.	0.2385	0.4884	100%
Omitting Cheng Jin ,2016	0.26	[0.25; 0.27]	.	0.2367	0.4865	100%
Omitting yang ze zheng,2023	0.26	[0.25; 0.27]	.	0.2377	0.4876	100%
Omitting chen guo ping,2024	0.26	[0.25; 0.27]	.	0.2386	0.4885	100%
Omitting Qin,Q.Y.2024	0.26	[0.25; 0.27]	.	0.2372	0.4870	100%
Omitting Zhao Hai ,2022	0.26	[0.25; 0.27]	.	0.2383	0.4881	100%
Omitting Zhao Jian Chao ,2022	0.26	[0.25; 0.27]	.	0.2385	0.4884	100%
Omitting He Min Mei ,2011	0.26	[0.25; 0.27]	.	0.2386	0.4885	100%
Omitting Li Xue ,2017	0.26	[0.25; 0.27]	.	0.2384	0.4882	100%
Omitting Yu Miao,2001	0.26	[0.25; 0.27]	.	0.2385	0.4884	100%
Omitting Zhuang Hong Juan ,2022	0.26	[0.25; 0.27]	.	0.2385	0.4883	100%
Omitting Hou Cheng Li ,2020	0.26	[0.25; 0.27]	.	0.2384	0.4883	100%
Omitting Hu Cai Xia ,2013	0.26	[0.25; 0.27]	.	0.2374	0.4872	100%
Omitting Wu Hong ,2015	0.26	[0.25; 0.27]	.	0.2365	0.4863	100%
Omitting Hou Yan Fei ,2018	0.26	[0.25; 0.27]	.	0.2375	0.4873	100%
Omitting Feng Jing Yao ,2020	0.26	[0.25; 0.27]	.	0.2382	0.4880	100%
Omitting Fan Rui Quan ,2007	0.26	[0.25; 0.27]	.	0.2383	0.4882	100%
Omitting Sun Yi ,2021	0.26	[0.25; 0.27]	.	0.2386	0.4885	100%
Omitting Xu, Y.,2016	0.26	[0.25; 0.27]	.	0.2386	0.4884	100%
Omitting mo hui zhen,2023	0.26	[0.25; 0.27]	.	0.2385	0.4883	100%
Omitting wu wen hua,2023	0.26	[0.25; 0.27]	.	0.2383	0.4882	100%
Omitting Zhong Ming Tian ,2012	0.26	[0.25; 0.27]	.	0.2381	0.4880	100%
Omitting Hao Xiao Ming ,1999	0.26	[0.25; 0.27]	.	0.2386	0.4884	100%
Omitting Li Long Long ,2018	0.26	[0.25; 0.27]	.	0.2384	0.4882	100%
Omitting Wei Ya Li ,2011	0.26	[0.25; 0.27]	.	0.2386	0.4884	100%
Omitting Lin Zhi Ping ,2001	0.26	[0.25; 0.27]	.	0.2385	0.4884	100%
Omitting Yu, X.F.2023	0.26	[0.25; 0.27]	.	0.2384	0.4882	100%
Omitting Li Shao Guang ,2007	0.26	[0.25; 0.27]	.	0.2368	0.4866	100%
Omitting Cheng Ran ,2012	0.26	[0.25; 0.27]	.	0.2369	0.4868	100%
Omitting Chen Yong Mei ,2021	0.26	[0.25; 0.27]	.	0.2378	0.4876	100%
Omitting Cai Xiao Meng ,2018	0.26	[0.25; 0.27]	.	0.2384	0.4883	100%
Omitting Zhang Xiao Juan ,2017	0.26	[0.25; 0.27]	.	0.2384	0.4883	100%
Omitting Guo, H.,2014	0.26	[0.25; 0.27]	.	0.2386	0.4885	100%
Omitting Xu, J.,2019	0.26	[0.25; 0.27]	.	0.2385	0.4883	100%
Omitting Bi Ye,2011	0.26	[0.25; 0.27]	.	0.2384	0.4883	100%
Omitting Ma Lan ,2020	0.26	[0.25; 0.27]	.	0.2377	0.4876	100%
Omitting Gong Yu Sha ,2020	0.26	[0.25; 0.27]	.	0.2367	0.4865	100%
Omitting Zhang Min Li ,2019	0.26	[0.25; 0.27]	.	0.2381	0.4879	100%
Omitting Ding, H.,2017	0.26	[0.25; 0.27]	.	0.2381	0.4879	100%
Omitting Chen Le Cheng ,2023	0.26	[0.25; 0.27]	.	0.2385	0.4883	100%
Omitting Jie Chao Ying ,2020	0.26	[0.25; 0.27]	.	0.2384	0.4883	100%
Omitting Liu Ai Min ,2014	0.26	[0.25; 0.27]	.	0.2386	0.4885	100%
Omitting Cao, R.,2020	0.26	[0.25; 0.27]	.	0.2386	0.4885	100%
Omitting Rao Yan Ting,2004	0.26	[0.25; 0.27]	.	0.2377	0.4876	100%
Omitting Yang Fan ,2022	0.26	[0.25; 0.27]	.	0.2383	0.4882	100%
Omitting Li Zhi Juan ,2022	0.26	[0.25; 0.27]	.	0.2386	0.4884	100%
Omitting tang biao qian,2024	0.26	[0.25; 0.27]	.	0.2385	0.4884	100%
Omitting He Zuo Li ,2021	0.26	[0.25; 0.27]	.	0.2381	0.4879	100%
Omitting Gao Qing ,2020	0.26	[0.25; 0.27]	.	0.2384	0.4883	100%
Omitting Li Mao Chun ,1999	0.26	[0.25; 0.27]	.	0.2385	0.4884	100%
Omitting Zhang Hong Ya ,2022	0.26	[0.25; 0.27]	.	0.2386	0.4885	100%
Omitting Dong, Y.,2020	0.26	[0.25; 0.27]	.	0.2385	0.4884	100%
Omitting Wang Shuai Fei ,2018	0.26	[0.25; 0.27]	.	0.2382	0.4880	100%
Omitting Shi, M.,2016	0.26	[0.25; 0.27]	.	0.2365	0.4864	100%
Omitting Fan Ai Ping ,2023	0.26	[0.25; 0.27]	.	0.2385	0.4884	100%
Omitting Wang Chao ,2023	0.26	[0.25; 0.27]	.	0.2386	0.4885	100%
Omitting Zhao Fu Cai ,2007	0.26	[0.25; 0.27]	.	0.2386	0.4885	100%
Omitting Zhao Jie ,2015	0.26	[0.25; 0.27]	.	0.2386	0.4884	100%
Omitting Liu Xia ,2021	0.26	[0.25; 0.27]	.	0.2385	0.4884	100%
Omitting Li Hai Feng ,2020	0.26	[0.25; 0.27]	.	0.2384	0.4883	100%
Omitting Ma Sheng Qi ,2020	0.26	[0.25; 0.27]	.	0.2384	0.4882	100%
Omitting Xu Li Ao ,2005	0.26	[0.25; 0.27]	.	0.2384	0.4882	100%
Omitting Li Shuang ,2006	0.26	[0.25; 0.27]	.	0.2371	0.4869	100%
Omitting Lai, S.2024	0.26	[0.25; 0.27]	.	0.2377	0.4875	100%
Omitting Zhang Yu Juan ,2006	0.26	[0.25; 0.27]	.	0.2378	0.4876	100%
Omitting Zhou Lin Lin ,2009	0.26	[0.25; 0.27]	.	0.2383	0.4882	100%
Omitting Shi Yong Bin ,2009	0.26	[0.25; 0.27]	.	0.2383	0.4882	100%
Omitting Jin Xia Fang ,2013	0.26	[0.25; 0.27]	.	0.2383	0.4881	100%
Omitting Shen Jin Bo ,2023	0.26	[0.25; 0.27]	.	0.2386	0.4885	100%
Omitting Ding Ji ,2023	0.26	[0.25; 0.27]	.	0.2386	0.4885	100%
Omitting Wu Jun Lan ,2020	0.26	[0.25; 0.27]	.	0.2365	0.4863	100%
Omitting Ge Sang Ze Ren ,2005	0.26	[0.25; 0.27]	.	0.2385	0.4884	100%
Omitting Yang Huai ,2013	0.26	[0.25; 0.27]	.	0.2381	0.4880	100%
Omitting Zou, S.K.2023	0.26	[0.25; 0.27]	.	0.2386	0.4884	100%
Omitting Wang Lei Xia ,2022	0.26	[0.25; 0.27]	.	0.2386	0.4885	100%
Omitting Chung, J.,2020	0.26	[0.25; 0.27]	.	0.2385	0.4883	100%
Omitting Ye, B.,2023	0.26	[0.25; 0.27]	.	0.2371	0.4870	100%
Omitting Xiamusiye Muyiduli,2024	0.26	[0.25; 0.27]	.	0.2372	0.4871	100%
Omitting Wu Hai Tan ,2023	0.26	[0.25; 0.27]	.	0.2383	0.4882	100%
Omitting Zhu Jian Ni ,2014	0.26	[0.25; 0.27]	.	0.2382	0.4881	100%
Omitting Chen Gui Cun ,2011	0.26	[0.25; 0.27]	.	0.2384	0.4883	100%
Omitting Jia Ming ,2012	0.26	[0.25; 0.27]	.	0.2386	0.4884	100%
Omitting Li Xue Mei ,2015	0.26	[0.25; 0.27]	.	0.2386	0.4884	100%
Omitting Shi Shan Shan ,2023	0.26	[0.25; 0.27]	.	0.2383	0.4881	100%
Omitting Zheng Li Feng ,2018	0.26	[0.25; 0.27]	.	0.2386	0.4884	100%
Omitting Zhang Cong Xiao ,2023	0.26	[0.25; 0.27]	.	0.2382	0.4881	100%
Omitting zhang cong xiao,2023	0.26	[0.25; 0.27]	.	0.2382	0.4881	100%
Omitting Lin, S. J.2023	0.26	[0.25; 0.27]	.	0.2384	0.4883	100%
Omitting Li, S.X.2024	0.26	[0.25; 0.27]	.	0.2379	0.4878	100%
Omitting Jiang, Q.,2023	0.26	[0.25; 0.27]	.	0.2380	0.4879	100%
Omitting Yan Na ,2022	0.26	[0.25; 0.27]	.	0.2385	0.4883	100%
Omitting mo min,2024	0.26	[0.25; 0.28]	.	0.2319	0.4815	100%
Omitting long yu,2024	0.26	[0.25; 0.27]	.	0.2386	0.4885	100%
Omitting Yu, Y.J.2023	0.26	[0.25; 0.27]	.	0.2375	0.4873	100%
Omitting Xu Xue Bing ,2015	0.26	[0.25; 0.27]	.	0.2386	0.4885	100%
Omitting Zhang Li ,2016	0.26	[0.25; 0.27]	.	0.2386	0.4884	100%
Omitting Cao Hui ,2012	0.26	[0.25; 0.27]	.	0.2386	0.4885	100%
Omitting Lai Yuan ,2012	0.26	[0.25; 0.27]	.	0.2384	0.4882	100%
Omitting Gu Jun Xia ,2007	0.26	[0.25; 0.27]	.	0.2383	0.4881	100%
Omitting Li Xiao Cai ,2010	0.26	[0.25; 0.27]	.	0.2384	0.4883	100%
Omitting Luo Kai ,2013	0.26	[0.25; 0.27]	.	0.2378	0.4876	100%
Omitting Su Lin Yan ,2006	0.26	[0.25; 0.27]	.	0.2382	0.4880	100%
Omitting Tang Jun,2003	0.26	[0.25; 0.27]	.	0.2382	0.4880	100%
Omitting zhang qin,2024	0.26	[0.25; 0.27]	.	0.2381	0.4880	100%
Omitting Yang Zhen Jiao ,2023	0.26	[0.25; 0.27]	.	0.2386	0.4884	100%
Omitting Zhang Hai Jun ,2017	0.26	[0.25; 0.27]	.	0.2385	0.4883	100%
Omitting Wang Kai ,2006	0.26	[0.25; 0.27]	.	0.2382	0.4880	100%
Omitting Dong Ying ,2021	0.26	[0.25; 0.27]	.	0.2376	0.4875	100%
Omitting Li Jin,2022	0.26	[0.25; 0.27]	.	0.2386	0.4884	100%
Omitting Li Guo ,2019	0.26	[0.25; 0.27]	.	0.2384	0.4883	100%
Omitting Liu Li Xin ,2016	0.26	[0.25; 0.27]	.	0.2385	0.4883	100%
Omitting Shen Zu De ,2006	0.26	[0.25; 0.27]	.	0.2378	0.4876	100%
Omitting Zhang Wen Wu ,2014	0.26	[0.25; 0.27]	.	0.2382	0.4880	100%
Omitting Tu Chun Yu ,2015	0.26	[0.25; 0.27]	.	0.2383	0.4882	100%
Omitting Xing Chao ,2016	0.26	[0.25; 0.27]	.	0.2383	0.4881	100%
Omitting Zhou Wei ,2015	0.26	[0.25; 0.27]	.	0.2373	0.4871	100%
Omitting Shen Yan ,2021	0.26	[0.25; 0.27]	.	0.2384	0.4883	100%
Omitting Meng Xiang Ning ,2016	0.26	[0.25; 0.27]	.	0.2386	0.4884	100%
Omitting Zhong Shu Fang ,2011	0.26	[0.25; 0.27]	.	0.2382	0.4881	100%
Omitting Yuan Guang Chao ,2022	0.26	[0.25; 0.27]	.	0.2385	0.4883	100%
Omitting Li Xiu Xia ,2019	0.26	[0.25; 0.27]	.	0.2378	0.4877	100%
Omitting Bao Min Ling ,2021	0.26	[0.25; 0.27]	.	0.2380	0.4878	100%
Omitting Xu Hai Ting,2019	0.26	[0.25; 0.27]	.	0.2381	0.4879	100%
Omitting Xu Ning ,2019	0.26	[0.25; 0.27]	.	0.2386	0.4884	100%
Omitting Ma Xiao Feng ,2020	0.26	[0.25; 0.27]	.	0.2386	0.4885	100%
Omitting Zhang, Y.F.2023	0.26	[0.25; 0.27]	.	0.2375	0.4873	100%
Omitting Li, J. H.,2019	0.26	[0.25; 0.27]	.	0.2373	0.4871	100%
Omitting Gu, Z.X.2024	0.26	[0.25; 0.27]	.	0.2383	0.4881	100%
Omitting yang rui jun,2024	0.26	[0.25; 0.27]	.	0.2375	0.4873	100%
Omitting He Li ,2023	0.26	[0.25; 0.27]	.	0.2381	0.4880	100%
Omitting Guo Jia Kun ,2022	0.26	[0.25; 0.27]	.	0.2366	0.4864	100%
Omitting Wang, W.2023	0.26	[0.25; 0.27]	.	0.2376	0.4875	100%
Omitting Zhou Jing Jing ,2018	0.26	[0.25; 0.27]	.	0.2385	0.4884	100%
Omitting Zhao Zi Han ,2023	0.26	[0.25; 0.27]	.	0.2378	0.4876	100%
Omitting Chi, G.,2022	0.26	[0.25; 0.27]	.	0.2378	0.4876	100%
Omitting Zhou, Y.2024	0.26	[0.25; 0.27]	.	0.2386	0.4885	100%
Omitting Xiao, R.,2020	0.26	[0.25; 0.27]	.	0.2380	0.4879	100%
Omitting Xiao Rong ,2017	0.26	[0.25; 0.27]	.	0.2385	0.4884	100%
Omitting Luo, J.2024	0.26	[0.25; 0.27]	.	0.2385	0.4883	100%
Omitting Qiu, L.2024	0.26	[0.25; 0.27]	.	0.2381	0.4879	100%
Omitting Lu Wen Ting,2023	0.26	[0.25; 0.27]	.	0.2386	0.4884	100%
Omitting Zeng, X.,2022	0.26	[0.25; 0.27]	.	0.2380	0.4878	100%
Omitting Liu, H.,2020	0.26	[0.25; 0.27]	.	0.2383	0.4882	100%
Omitting liu rui jie,2023	0.26	[0.25; 0.27]	.	0.2381	0.4879	100%
Omitting kong ling hua,2023	0.26	[0.25; 0.27]	.	0.2383	0.4881	100%
Omitting Luo M.M.2024	0.26	[0.25; 0.27]	.	0.2377	0.4875	100%
Omitting Li Xin Xin ,2022	0.26	[0.25; 0.27]	.	0.2371	0.4869	100%
Omitting sun zheng hai,2023	0.26	[0.25; 0.27]	.	0.2386	0.4884	100%
Omitting Yong,Z.T.2024	0.26	[0.25; 0.27]	.	0.2381	0.4879	100%
Omitting Liu Yang ,2017	0.26	[0.25; 0.27]	.	0.2382	0.4880	100%
Omitting Wu Zhi Hong ,2023	0.26	[0.25; 0.27]	.	0.2382	0.4881	100%
Omitting Yang, M.Y.2023	0.26	[0.25; 0.27]	.	0.2386	0.4885	100%
Omitting Wang, Y.2023	0.26	[0.25; 0.27]	.	0.2375	0.4874	100%
Omitting Doris?Y.?P.?Leung,2023	0.26	[0.25; 0.27]	.	0.2348	0.4846	100%
Omitting Zhang Zhe Dong ,2023	0.26	[0.25; 0.27]	.	0.2376	0.4875	100%
Omitting Lv,X.H,2023	0.26	[0.25; 0.27]	.	0.2382	0.4881	100%
Omitting Wang, H.,2023	0.26	[0.25; 0.27]	.	0.2385	0.4884	100%
Omitting Peng, P.,2023	0.26	[0.25; 0.27]	.	0.2383	0.4881	100%
Omitting Lv,X.H,2023	0.26	[0.25; 0.27]	.	0.2384	0.4882	100%
Omitting Peng, P.2023	0.26	[0.25; 0.27]	.	0.2368	0.4866	100%
Omitting Wang, H.2023	0.26	[0.25; 0.27]	.	0.2385	0.4884	100%
Omitting Jin, Z.2024	0.26	[0.25; 0.27]	.	0.2385	0.4884	100%
Omitting Li, S.Q.2024	0.26	[0.25; 0.27]	.	0.2380	0.4878	100%
Omitting Yang Yan Fang ,2012	0.26	[0.25; 0.27]	.	0.2385	0.4884	100%
Omitting Wang, Q.,2022	0.26	[0.25; 0.27]	.	0.2386	0.4885	100%
Omitting Guo Kai ,2014	0.26	[0.25; 0.27]	.	0.2374	0.4872	100%
Omitting Cui Xiang Rui ,1988	0.26	[0.25; 0.27]	.	0.2385	0.4883	100%
Omitting Ye Lei ,2016	0.26	[0.25; 0.27]	.	0.2385	0.4884	100%
Omitting Zeng Zhi Fa ,2013	0.26	[0.25; 0.27]	.	0.2384	0.4883	100%
Omitting Yang Rui ,2008	0.26	[0.25; 0.27]	.	0.2378	0.4876	100%
Omitting Li Tian Tian ,2016	0.26	[0.25; 0.27]	.	0.2383	0.4882	100%
Omitting Zhao Shu Juan ,2009	0.26	[0.25; 0.27]	.	0.2385	0.4883	100%
Omitting Fan Chun Ling ,2007	0.26	[0.25; 0.27]	.	0.2348	0.4846	100%
Omitting Yang Xiu Lan ,2015	0.26	[0.25; 0.27]	.	0.2358	0.4856	100%
Omitting Zhang Zhi Jun ,2000	0.26	[0.25; 0.27]	.	0.2378	0.4876	100%
Omitting Li Ying Chun ,2005	0.26	[0.25; 0.27]	.	0.2385	0.4884	100%
Omitting Wang Da Zhu ,2019	0.26	[0.25; 0.27]	.	0.2386	0.4884	100%
Omitting Rong Zhi ,2020	0.26	[0.25; 0.27]	.	0.2386	0.4885	100%
Omitting Wang Jun ,2013	0.26	[0.25; 0.27]	.	0.2386	0.4885	100%
Omitting Cui Ran ,2013	0.26	[0.25; 0.27]	.	0.2386	0.4885	100%
Omitting Gu Fei Fei ,2012	0.26	[0.25; 0.27]	.	0.2386	0.4885	100%
Omitting Yu Jie Gen ,2015	0.26	[0.25; 0.27]	.	0.2386	0.4884	100%
Omitting Qu Wei ,2014	0.26	[0.25; 0.27]	.	0.2385	0.4884	100%
Omitting Chu Bin Lin ,2013	0.26	[0.25; 0.27]	.	0.2385	0.4884	100%
Omitting Jiang De Qin ,2011	0.26	[0.25; 0.27]	.	0.2385	0.4884	100%
Omitting Wang Hui ,2013	0.26	[0.25; 0.27]	.	0.2385	0.4884	100%
Omitting Zheng Rui Zhi ,2012	0.26	[0.25; 0.27]	.	0.2385	0.4883	100%
Omitting Tian Zhen Shang ,2007	0.26	[0.25; 0.27]	.	0.2384	0.4883	100%
Omitting Tang Jian Jun ,2009	0.26	[0.25; 0.27]	.	0.2384	0.4883	100%
Omitting Ni Shi Gang ,2012	0.26	[0.25; 0.27]	.	0.2384	0.4882	100%
Omitting He ting ting,2020	0.26	[0.25; 0.27]	.	0.2383	0.4882	100%
Omitting Lu Jing Ru ,2021	0.26	[0.25; 0.27]	.	0.2383	0.4882	100%
Omitting Tang Hui ,2013	0.26	[0.25; 0.27]	.	0.2381	0.4880	100%
Omitting Han Shi Yu,2020	0.26	[0.25; 0.27]	.	0.2381	0.4880	100%
Omitting Li Xin ,2018	0.26	[0.25; 0.27]	.	0.2377	0.4876	100%
Omitting Gao Jie ,2016	0.26	[0.25; 0.27]	.	0.2377	0.4875	100%
Omitting He Chun Ling ,2020	0.26	[0.25; 0.27]	.	0.2367	0.4865	100%
Omitting Lu Chen ,2021	0.26	[0.25; 0.27]	.	0.2385	0.4884	100%
Omitting Chen Jing Yi ,2017	0.26	[0.25; 0.27]	.	0.2376	0.4875	100%
Omitting Wang Li Xin,2016	0.26	[0.25; 0.27]	.	0.2386	0.4884	100%
Omitting Li Ying Tao ,2020	0.26	[0.25; 0.27]	.	0.2386	0.4884	100%
Omitting Chen Lin Lin ,2016	0.26	[0.25; 0.27]	.	0.2386	0.4885	100%
Omitting He Hong ,2015	0.26	[0.25; 0.27]	.	0.2385	0.4883	100%
Omitting Wang, C.2023	0.26	[0.25; 0.27]	.	0.2382	0.4881	100%
Omitting Ma,Y.X.2024	0.26	[0.25; 0.27]	.	0.2385	0.4884	100%
Omitting Ji Dong ,2018	0.26	[0.25; 0.27]	.	0.2370	0.4869	100%
Omitting Li You La ,2011	0.26	[0.25; 0.27]	.	0.2385	0.4884	100%
Omitting Li Ying Di,2021	0.26	[0.25; 0.27]	.	0.2375	0.4873	100%
Omitting Liu Chang Jie ,2007	0.26	[0.25; 0.27]	.	0.2366	0.4864	100%
Omitting Liu Meng ,2019	0.26	[0.25; 0.27]	.	0.2385	0.4883	100%
Omitting Lai Qiao Zhen ,2018	0.26	[0.25; 0.27]	.	0.2364	0.4862	100%
Omitting Zeng Lin Na ,2003	0.26	[0.25; 0.27]	.	0.2376	0.4875	100%
Omitting Yuan Ding ,2013	0.26	[0.25; 0.27]	.	0.2379	0.4878	100%
Omitting Wang Meng Long ,2003	0.26	[0.25; 0.27]	.	0.2385	0.4884	100%
Omitting Liu Jian An ,2003	0.26	[0.25; 0.27]	.	0.2386	0.4884	100%
Omitting Luo Li Fen ,2019	0.26	[0.25; 0.27]	.	0.2386	0.4885	100%
Omitting Hou Qiang ,2008	0.26	[0.25; 0.27]	.	0.2386	0.4885	100%
Omitting Ma Ze Wei ,2013	0.26	[0.25; 0.27]	.	0.2386	0.4884	100%
Omitting Huang Juan Xu ,2008	0.26	[0.25; 0.27]	.	0.2385	0.4884	100%
Omitting Yang Huan Cheng ,2019	0.26	[0.25; 0.27]	.	0.2382	0.4880	100%
Omitting Wang Man ,2015	0.26	[0.25; 0.27]	.	0.2381	0.4880	100%
Omitting Bai Lu Xi,2012	0.26	[0.25; 0.27]	.	0.2381	0.4880	100%
Omitting Lin Hua Fen ,2012	0.26	[0.25; 0.27]	.	0.2372	0.4871	100%
Omitting Deng Hai Ling ,2019	0.26	[0.25; 0.27]	.	0.2381	0.4880	100%
Omitting Dong Xiu Yun ,2017	0.26	[0.25; 0.27]	.	0.2380	0.4878	100%
Omitting lu wen fang,2024	0.26	[0.25; 0.27]	.	0.2383	0.4882	100%
Omitting Li Ya Fei ,2009	0.26	[0.25; 0.27]	.	0.2377	0.4876	100%
Omitting Li Yan ,2008	0.26	[0.25; 0.27]	.	0.2386	0.4885	100%
Omitting Kang Xiao Hua ,2010	0.26	[0.25; 0.27]	.	0.2385	0.4884	100%
Omitting Lan Jie ,2017	0.26	[0.25; 0.27]	.	0.2384	0.4883	100%
Omitting Guo Rong ,2005	0.26	[0.25; 0.27]	.	0.2382	0.4880	100%
Omitting Jia Pei Ling ,2021	0.26	[0.25; 0.27]	.	0.2386	0.4885	100%
Omitting Feng Feng Lian ,2018	0.26	[0.25; 0.27]	.	0.2375	0.4873	100%
Omitting Ma Hui ,2019	0.26	[0.25; 0.27]	.	0.2380	0.4879	100%
Omitting Xi Ming Jing ,2010	0.26	[0.25; 0.27]	.	0.2385	0.4883	100%
Omitting Gao Di ,2019	0.26	[0.25; 0.27]	.	0.2386	0.4884	100%
Omitting Zhao Bin ,2015	0.26	[0.25; 0.27]	.	0.2383	0.4882	100%
Omitting Jing Kun Juan ,2011	0.26	[0.25; 0.27]	.	0.2378	0.4877	100%
Omitting bai mei ling,2023	0.26	[0.25; 0.27]	.	0.2385	0.4884	100%
Omitting Wu Guo Lan ,2013	0.26	[0.25; 0.27]	.	0.2380	0.4879	100%
Omitting Bai Sheng Hao ,2007	0.26	[0.25; 0.27]	.	0.2382	0.4881	100%
Omitting Wang Wen Xia ,2008	0.26	[0.25; 0.27]	.	0.2382	0.4881	100%
Omitting Wei Jun Biao ,2006	0.26	[0.25; 0.27]	.	0.2386	0.4885	100%
Omitting Zhao Chuan ,2015	0.26	[0.25; 0.27]	.	0.2385	0.4884	100%
Omitting Shi Shen Chao ,2012	0.26	[0.25; 0.27]	.	0.2372	0.4870	100%
Omitting Li Yu Xia ,2013	0.26	[0.25; 0.27]	.	0.2361	0.4859	100%
Omitting Zhao Xi Wu ,2015	0.26	[0.25; 0.27]	.	0.2385	0.4884	100%
Omitting Ren Xiao Hui ,2013	0.26	[0.25; 0.27]	.	0.2386	0.4884	100%
Omitting Xu Li Li ,2010	0.26	[0.25; 0.27]	.	0.2386	0.4884	100%
Omitting Ma bao chen,2016	0.26	[0.25; 0.27]	.	0.2386	0.4885	100%
Omitting Wang Jing Jing ,2007	0.26	[0.25; 0.27]	.	0.2385	0.4883	100%
Omitting Yao Qi He ,1994	0.26	[0.25; 0.27]	.	0.2380	0.4879	100%
Omitting Yuan, Y.2024	0.26	[0.25; 0.27]	.	0.2344	0.4841	100%
Omitting Qin Tai Ran ,2007	0.26	[0.25; 0.27]	.	0.2373	0.4871	100%
Omitting Chai Xiao Rong ,2011	0.26	[0.25; 0.27]	.	0.2386	0.4884	100%
Omitting Wang Zu Ti ,2007	0.26	[0.25; 0.27]	.	0.2386	0.4884	100%
Omitting Xu Ying ,2003	0.26	[0.25; 0.27]	.	0.2386	0.4884	100%
Omitting Zheng Shi Hua ,2016	0.26	[0.25; 0.27]	.	0.2385	0.4884	100%
Omitting Xiong Ai Jiao,2007	0.26	[0.25; 0.27]	.	0.2384	0.4883	100%
Omitting Ye Zhi Ying ,2013	0.26	[0.25; 0.27]	.	0.2384	0.4883	100%
Omitting Hu Su Yun ,2021	0.26	[0.25; 0.27]	.	0.2384	0.4882	100%
Omitting Zhong Yi ,2007	0.26	[0.25; 0.27]	.	0.2382	0.4881	100%
Omitting Mou Su Hua ,1999	0.26	[0.25; 0.27]	.	0.2380	0.4879	100%
Omitting Wang Kai ,2015	0.26	[0.25; 0.27]	.	0.2379	0.4877	100%
Omitting Chen Xiao Xuan,2017	0.26	[0.25; 0.27]	.	0.2385	0.4884	100%
Omitting Tian Sha ,2019	0.26	[0.25; 0.27]	.	0.2385	0.4884	100%
Omitting Chen Si Si ,2020	0.26	[0.25; 0.27]	.	0.2386	0.4885	100%
Omitting Tang, Z.,2021	0.26	[0.25; 0.27]	.	0.2386	0.4884	100%
Omitting Hou Yu Qi,2020	0.26	[0.25; 0.27]	.	0.2384	0.4882	100%
Omitting He Xin Yun ,2013	0.26	[0.25; 0.27]	.	0.2375	0.4873	100%
Omitting Jiang Zhi Yong ,2014	0.26	[0.25; 0.27]	.	0.2375	0.4873	100%
Omitting Zhang Jing Ping ,2013	0.26	[0.25; 0.27]	.	0.2374	0.4872	100%
Omitting Zhong, Y.,2021	0.26	[0.25; 0.27]	.	0.2385	0.4884	100%
Omitting Li Xuan Xuan ,2018	0.26	[0.25; 0.27]	.	0.2386	0.4884	100%
Omitting Huo Bin ,2010	0.26	[0.25; 0.27]	.	0.2386	0.4885	100%
Omitting Li Chun Jie ,2014	0.26	[0.25; 0.27]	.	0.2382	0.4881	100%
Omitting Wang Jin Yang ,2016	0.26	[0.25; 0.27]	.	0.2381	0.4880	100%
Omitting Fu Gui Fang ,2010	0.26	[0.25; 0.27]	.	0.2376	0.4874	100%
Omitting Yang Xue Hua ,2007	0.26	[0.25; 0.28]	.	0.2283	0.4778	100%
Omitting Ye Hong Ping ,2015	0.26	[0.25; 0.27]	.	0.2384	0.4883	100%
Omitting Lv Ding Ding ,2013	0.26	[0.25; 0.27]	.	0.2386	0.4884	100%
Omitting Qian Yun Ke ,2016	0.26	[0.25; 0.27]	.	0.2385	0.4884	100%
Omitting Jin Peng ,2014	0.26	[0.25; 0.27]	.	0.2384	0.4882	100%
Omitting Shen Yi ,2007	0.26	[0.25; 0.27]	.	0.2381	0.4880	100%
Omitting Hong Xin ,2011	0.26	[0.25; 0.27]	.	0.2380	0.4878	100%
Omitting Yang Xue ,2018	0.26	[0.25; 0.27]	.	0.2380	0.4878	100%
Omitting Yu Jie ,2004	0.26	[0.25; 0.27]	.	0.2379	0.4878	100%
Omitting Qi Sheng Xiang ,2012	0.26	[0.25; 0.27]	.	0.2379	0.4877	100%
Omitting Sun Zhi Qing ,2012	0.26	[0.25; 0.27]	.	0.2379	0.4877	100%
Omitting Zhou Hai Rong ,2015	0.26	[0.25; 0.27]	.	0.2379	0.4877	100%
Omitting Lv Huai Juan ,2012	0.26	[0.25; 0.27]	.	0.2379	0.4877	100%
Omitting Chen Tong Qing ,2011	0.26	[0.25; 0.27]	.	0.2378	0.4876	100%
Omitting Shen Su Mei ,2022	0.26	[0.25; 0.27]	.	0.2370	0.4868	100%
Omitting Wang Ning Xia ,2010	0.26	[0.25; 0.27]	.	0.2386	0.4885	100%
Omitting Zhang Si Ming ,2010	0.26	[0.25; 0.27]	.	0.2384	0.4883	100%
Omitting Zhang Rong Hua ,2012	0.26	[0.25; 0.27]	.	0.2380	0.4878	100%
Omitting Xu Li Mei ,2002	0.26	[0.25; 0.27]	.	0.2379	0.4878	100%
Omitting Zhang, Z.,2019	0.26	[0.25; 0.27]	.	0.2385	0.4883	100%
Omitting Guo, F.,2020	0.26	[0.25; 0.27]	.	0.2385	0.4884	100%
Omitting Di De Chun ,2005	0.26	[0.25; 0.27]	.	0.2385	0.4884	100%
Omitting Jin Zhe,2006	0.26	[0.25; 0.27]	.	0.2385	0.4884	100%
Omitting Yang, Y.2023	0.26	[0.25; 0.27]	.	0.2385	0.4883	100%
Omitting Ma Qian Min,2023	0.26	[0.25; 0.27]	.	0.2378	0.4876	100%
Omitting Wu Shi Zhen ,2016	0.26	[0.25; 0.27]	.	0.2363	0.4862	100%
Omitting Liu, X. C.,1999	0.26	[0.25; 0.27]	.	0.2381	0.4880	100%
Omitting Zhang Feng Mei ,2014	0.26	[0.25; 0.27]	.	0.2384	0.4882	100%
Omitting Niu Rong Hua ,2010	0.26	[0.25; 0.27]	.	0.2385	0.4884	100%
Omitting Cheng, S.,2020	0.26	[0.25; 0.27]	.	0.2385	0.4884	100%
Omitting Yu Xiang Fen ,2011	0.26	[0.25; 0.27]	.	0.2386	0.4884	100%
Omitting Wei Qiu Xiang ,1998	0.26	[0.25; 0.27]	.	0.2386	0.4885	100%
Omitting Jiang Shun Li ,2022	0.26	[0.25; 0.27]	.	0.2386	0.4884	100%
Omitting Cui Qing Xia ,2014	0.26	[0.25; 0.27]	.	0.2385	0.4884	100%
Omitting Qian Shu Yan ,2022	0.26	[0.25; 0.27]	.	0.2384	0.4883	100%
Omitting Li Jing ,1997	0.26	[0.25; 0.27]	.	0.2370	0.4869	100%
Omitting dong gui xia,2023	0.26	[0.25; 0.27]	.	0.2384	0.4883	100%
Omitting Wu Hua Lin ,2000	0.26	[0.25; 0.27]	.	0.2380	0.4879	100%
Omitting Gu Wen Yi ,2009	0.26	[0.25; 0.27]	.	0.2386	0.4885	100%
Omitting Che Bei Bei ,2021	0.26	[0.25; 0.27]	.	0.2384	0.4882	100%
Omitting Xu Hao ,2013	0.26	[0.25; 0.27]	.	0.2386	0.4884	100%
Omitting Dai Cheng Shu ,2011	0.26	[0.25; 0.27]	.	0.2380	0.4879	100%
Omitting Yi Xiao Ming ,2015	0.26	[0.25; 0.27]	.	0.2377	0.4875	100%
Omitting Zhang Qian Ying ,2023	0.26	[0.25; 0.27]	.	0.2368	0.4866	100%
Omitting Wang Zhen Xiang ,2019	0.26	[0.25; 0.27]	.	0.2384	0.4883	100%
Omitting Yang, Y.2024	0.26	[0.25; 0.27]	.	0.2380	0.4879	100%
Omitting Qu Ming ,2018	0.26	[0.25; 0.27]	.	0.2384	0.4883	100%
Omitting Su Yun Peng ,2018	0.26	[0.25; 0.27]	.	0.2386	0.4884	100%
Omitting Li Li ,2004	0.26	[0.25; 0.27]	.	0.2365	0.4863	100%
Omitting Zeng Rui Hua ,2010	0.26	[0.25; 0.27]	.	0.2377	0.4875	100%
Omitting Zhou Bo ,2018	0.26	[0.25; 0.27]	.	0.2382	0.4881	100%
Omitting Zhang Xiu Juan ,2006	0.26	[0.25; 0.27]	.	0.2384	0.4883	100%
Omitting Zhang Yong ,2020	0.26	[0.25; 0.27]	.	0.2384	0.4883	100%
Omitting Zhu Ge Yi ,2007	0.26	[0.25; 0.27]	.	0.2385	0.4883	100%
Omitting Li Meng Yao ,2021	0.26	[0.25; 0.27]	.	0.2386	0.4885	100%
Omitting Dong Dan Dan ,2022	0.26	[0.25; 0.27]	.	0.2386	0.4885	100%
Omitting Tu Chun Yu ,2011	0.26	[0.25; 0.27]	.	0.2381	0.4879	100%
Omitting Xing Chao ,2011	0.26	[0.25; 0.27]	.	0.2381	0.4879	100%
Omitting shen lian xiang,2024	0.26	[0.25; 0.27]	.	0.2371	0.4870	100%
Omitting Han Li ,2014	0.26	[0.25; 0.27]	.	0.2373	0.4871	100%
Omitting Gong Juan ,2020	0.26	[0.25; 0.27]	.	0.2386	0.4885	100%
Omitting Hao Li nan,2011	0.26	[0.25; 0.27]	.	0.2383	0.4881	100%
Omitting Wang Hong ,2009	0.26	[0.25; 0.27]	.	0.2386	0.4885	100%
Omitting Zhang Li ,2003	0.26	[0.25; 0.27]	.	0.2385	0.4884	100%
Omitting Chen Xiao Qin ,2011	0.26	[0.25; 0.27]	.	0.2384	0.4883	100%
Omitting Liao Quan Ming ,2010	0.26	[0.25; 0.27]	.	0.2384	0.4883	100%
Omitting Zheng Han Feng ,2015	0.26	[0.25; 0.27]	.	0.2380	0.4879	100%
Omitting Shao, R.,2020	0.26	[0.25; 0.27]	.	0.2372	0.4870	100%
Omitting Li Lei Lei ,2010	0.26	[0.25; 0.27]	.	0.2371	0.4869	100%
Omitting Shao, R.Y,2023	0.26	[0.25; 0.27]	.	0.2375	0.4873	100%
Omitting Feng Zheng Zhi ,2005	0.26	[0.25; 0.27]	.	0.2381	0.4879	100%
Omitting Liu Xian Chen ,1997	0.26	[0.25; 0.27]	.	0.2381	0.4880	100%
**Random effects model**	**0.26**	**[0.25; 0.27]**	**.**	**0.2380**	**0.4879**	**100%**

−0.2 −0.1 0 0.1 0.2
