# Supplementary material for: Prevalence of depressive symptoms among children and adolescents in china: a systematic review and meta-analysis
Source: Child Adolesc Psychiatry Ment Health. 2024 Nov 19;18:150. doi: 10.1186/s13034-024-00841-w (PMC11577650; doi:10.1186/s13034-024-00841-w)
Supplement: Supplementary file 4 — Additional file 4. [file 13034_2024_841_MOESM4_ESM.pdf]

Supplementary Tables

Table S1 Prevalence of depressive symptoms of children and adolescents in different years

Year	Studies	Sample	Proportion [95%CI]	I^2
1988	1	219	0.2100 [0.1625; 0.2716]	--
1991	1	335	0.4806 [0.4300; 0.5372]	--
1994	1	237	0.4304 [0.3717; 0.4983]	--
1997	2	3232	0.3168 [0.0925; 1.0000]	100%
1998	1	312	0.2596 [0.2153; 0.3131]	--
1999	6	6178	0.2764 [0.2122; 0.3600]	98%
2000	3	2877	0.3244 [0.1458; 0.7217]	98%
2001	4	15531	0.2543 [0.2081; 0.3109]	93%
2002	2	717	0.2505 [0.0901; 0.6960]	97%
2003	7	3593	0.2396 [0.1754; 0.3273]	98%
2004	4	8544	0.1999 [0.1057; 0.3781]	100%
2005	8	9052	0.3362 [0.2825; 0.4001]	95%
2006	9	13220	0.2205 [0.1550; 0.3138]	99%
2007	24	39179	0.2348 [0.1767; 0.3119]	100%
2008	7	4912	0.2799 [0.1869; 0.4192]	99%
2009	14	36234	0.2267 [0.1859; 0.2766]	99%
2010	14	8894	0.2892 [0.2374; 0.3523]	98%
2011	21	36964	0.3010 [0.2552; 0.3549]	99%
2012	19	36929	0.3039 [0.2401; 0.3847]	99%
2013	25	42715	0.2865 [0.2354; 0.3486]	100%
2014	16	23203	0.2949 [0.2360; 0.3684]	99%
2015	23	53980	0.2505 [0.2017; 0.3111]	99%
2016	22	60929	0.2740 [0.2264; 0.3316]	100%
2017	14	28359	0.2359 [0.1850; 0.3008]	99%
2018	22	81348	0.2103 [0.1718; 0.2575]	100%
2019	19	43880	0.2612 [0.2185; 0.3124]	99%
2020	31	95728	0.2572 [0.2211; 0.2992]	99%
2021	19	72177	0.2620 [0.2215; 0.3097]	99%
2022	21	49050	0.2940 [0.2452; 0.3524]	100%
2023	51	365952	0.2653 [0.2287; 0.3079]	100%
2024	28	353044	0.2236 [0.1782; 0.2805]	100%

Note: Test for subgroup differences (random effects): $\chi 2==158.69$, *(*$p < 0.01$*)*

Table S2 Prevalence of depressive symptoms of children and adolescents in provinces and municipalities

Province	Studies	Sample	Proportion [95%CI]	I^2
Anhui	49	239972	27.85 [24.17-32.10]	100%
Beijing	21	42071	26.89 [22.17-32.61]	100%
Chongqing	17	51868	27.86 [20.81-37.30]	100%
Fujian	6	7362	39.55 [30.49-51.31]	99%
Gansu	4	10659	24.74 [15.63-39.16]	97%
Guangdong	33	90356	21.75 [18.70-25.31]	99%
Guangxi	4	3214	38.00 [32.40-44.57]	88%
Guizhou	10	32639	28.55 [23.52-34.67]	98%
Hainan	4	14020	25.99 [19.00-35.54]	98%
Hebei	11	16060	26.18 [19.70-34.80]	99%
Heilongjiang	12	21846	22.97 [18.48-28.57]	98%
Henan	19	31960	26.03 [20.54-32.98]	100%
Hong Kong	6	13345	27.06 [14.52-50.44]	100%
Hubei	19	39473	23.24 [18.48-29.21]	100%
Hunan	18	32791	24.07 [19.32-29.98]	100%
Jiangsu	26	145143	25.96 [20.14-33.46]	100%
Jiangxi	4	2317	37.46 [28.35-49.49]	96%
Jilin	6	7165	33.34 [25.95-42.84]	98%
Liaoning	14	56759	26.40 [20.93-33.30]	100%
Inner Mongolia	3	6242	18.43 [11.98-28.36]	98%
Ningxia	3	2162	31.85 [24.17-41.99]	92%
Qinghai	1	454	54.19 [49.79-58.97]	--
Shaanxi	9	13624	29.28 [22.17-38.67]	99%
Shandong	28	41006	27.44 [23.49-32.05]	99%
Shanghai	18	53420	22.34 [17.69-28.21]	100%
Shanxi	10	39626	26.29 [17.57-39.34]	100%
Sichuan	13	26680	30.67 [23.60-39.86]	99%
Taiwan	1	625	32.64 [29.16-36.53]	--
Xinjiang	8	43035	20.35 [13.61-30.42]	100%
Tibet	2	10584	47.50 [39.99-56.42]	99%
Yunnan	8	45178	25.41 [21.08-30.64]	99%
Zhejiang	24	112564	23.89 [19.85-28.74]	100%
Tianjin	1	9185	31.52 [30.58-32.48]	--

Note: No study conducted solely in Tianjin was included, the data of Tianjin was from a study[1] in which Tianjin was one of the important study sites. Test for subgroup differences (random effects): $\chi 2 = 288.64$, $(p < 0.01)$

[1] Lu, C., Y. Zhu, C. Zhan, J. Li, J. Zhu, and B. Li, *Study on the relationship between depression and peer support in Chinese medical colleges and universities.* Chinese Journal of Health Education, 2021. **37**(02): 153-156.
